# Comparison of Salt-Related Knowledge and Behaviors Status of WeChat Users between 2019 and 2020

**DOI:** 10.3390/nu13072141

**Published:** 2021-06-22

**Authors:** Yibing Yang, Jinglei Wang, Jixiang Ma, Wenhui Shi, Jing Wu

**Affiliations:** 1 Office of Non-Communicable Disease and Aging Health Management, Chinese Center for Disease Control and Prevention, Beijing 102206, China; yangyb@chinacdc.cn (Y.Y.); wangjl100@chinacdc.cn (J.W.); 2 Shandong Center for Disease Control and Prevention, Jinan 250014, China; majix@163.com; 3 National Center for Chronic and Noncommunicable Disease Control and Prevention, Chinese Center for Disease Control and Prevention, Beijing 100050, China

**Keywords:** salt reduction, salt-related knowledge, high salt intake, China Healthy Lifestyle for All

## Abstract

In order to identify the status of salt-related knowledge and behavior of the residents who were active in WeChat software between 2019 and 2020, 10-day salt-related surveys were conducted in 2019 and 2020 based on the WeChat public platform of China Healthy Lifestyle for All Campaign. Distribution and scores of salt-related knowledge, salt reduction behavior and high-salt intake behavior between 2019 and 2020 were compared. Data of 2109 participants in 2019 and 12,732 participants in 2020 were left for analysis. Overall, 88.2% of participants in 2019 had a willingness to reduce the amount of cooking salt in their households, significantly lower than 90.2% in 2020 (*p*-value < 0.05). In 2019 and 2020, over 80% of the participants knew fine dried noodles contain salt, but less than 30% knew ice cream contains salt. Over 78% of participants chose 5 g or 6 g for the maximum daily salt intake of healthy adults, and about 98% of participants knew that excessive salt intake would increase the risk of hypertension in both years. The percentage of participants who used salt measuring spoons asked restaurants to use less salt, read the sodium content on the nutrition facts table, chose foods with low sodium content and regularly used low-sodium salt, were 36.1%, 45.0%, 44.1%, 40.3% and 35.8% in 2019, and the percentage increased significantly to 46.4%, 49.2%, 50.8%, 47.1% and 43.4% in 2020 (all *p*-value < 0.05). The percentage of people regularly eating pickled mustard tubers, salted vegetables and sauce foods or using high-salt condiments also increased from 2019 to 2020. The median of salt-related knowledge scores, salt reduction behavior scores and high-salt intake behavior scores were 11, 2, 5 points in 2019, and 10, 3, 5 points in 2020, respectively. Compared to 2019, the salt-related knowledge score was relatively lower, while the salt reduction behavior score and high-salt intake behavior score were relatively higher in 2020. Besides, the score of salt-related knowledge and behaviors differed in different gender, age and hypertension groups. The COVID-19 epidemic may have influenced the salt-related knowledge and behaviors status of WeChat users in China. Promotion and education of salt-related knowledge and online behavior intervention are still needed, particularly for male and hypertension patients in the future.

## 1. Introduction

Non-communicable diseases have become a huge threat to the lives and health of the global public; the onset and long-term treatment of chronic diseases will not only reduce the quality of life of the patients and their families but also cause society to rapidly increase in huge expenditures and bear heavy burdens [1,2,3]. Ischemic heart disease and stroke had become the top-ranked causes of disability adjusted life years(DALYs) in both the 50–74-year and 75-years-and-older age groups across the world, and it was estimated that hypertension and cardiovascular diseases accounted for more than 40% of all deaths in China and were the main causes of all deaths [2,4]. The excessive intake of salt may increase the risk of hypertension, cardiovascular disease and stroke [5,6,7]. Over 3 million deaths worldwide were related to a diet high in sodium in 2017 [8]. Reducing salt intake has been identified as one of the most cost-effective measures countries can take to improve population health outcomes, and if the global salt consumption was reduced to the recommended level, millions of deaths could be prevented [9]. However, the salt intakes of residents of China were still very high, the average salt intake was 9.3 g/day from the latest report [10], and the average sodium intake was 4318.1 ± 1814.1 mg/d (equivalent to 11.0 ± 4.6 g/day of salt) in six provinces of the Action on Salt China in 2018 [11], which were both higher than the recommended maximum level of intake by WHO (5 g/day) [12] and Chinese Nutrition Society (6 g/day) [13]. 

In order to raise public awareness of salt reduction and promote salt reduction behaviors, many action plans and studies had been launched in the mainland of China in the past few decades [11,14,15,16]. China Healthy Lifestyle for All Campaign (CHLA) was initiated in 2007; CHLA stage one was from 2007 to 2015 with the theme, “Harmonious life, and healthy Chinese people”, and all kinds of easy-to-use health support tools such as a salt-restricted spoon, oil control pot and body mass index calculation ruler were developed and promoted [17]. In stage two (2017–2025), the content of CHLA was further expanded. “Three reduction, three healthy” actions including salt reduction, oil reduction, sugar reduction, healthy oral cavity, healthy weight and healthy bones was the first action of CHLA. Salt-reduction-related promotional materials such as audio, video, poster and graphics context materials were developed by the national action office of the CHLA in the past few years and promoted to the whole country for online and offline health education activities. A salt-reduction-related conference was held and an initiative was released [18]. A previous study had found that participants in “CHLA intervention” counties were more likely to know the limits of salt and were more intent to modify their salt consumption [19].

Education on salt-related knowledge and how to reduce salt intake could reduce 24 h urinary sodium of children and their families from the School-EduSalt study, and better diet-related knowledge, attitudes and behaviors were associated with higher health status [16,20]. However, behavior change is complex, long-term and slow; understanding the current population statuses of salt-related knowledge, attitudes and behaviors are essential for further salt-reduction actions [21], and similar studies had been conducted in Australia, America and other countries [22,23,24,25,26]. Salt-related knowledge and behaviors of Chinese residents in 2015 were explored in a previous study [27], based on the data of data from the China Chronic Diseases and Nutrition Surveillance system, and low awareness rate of maximum daily salt intake and the behavior rate of salt reduction of Chinese adult residents were found. However, in recent years, much more activities in the publicity and advocacy of salt reduction have been carried out by the National Health Commission of China, the World Health Organization, the national action office of CHLA and other institutions [18,28,29,30]. In 2019, the Bureau of Disease Control and Prevention of the National Health Commission launched the “9.15” Salt Reduction Publicity Week, and all regions of the country actively responded and carried out a large number of salt-reduction-related activities [28]. Besides, the unexpected coronavirus disease 2019 (COVID-19) epidemic across the whole country in early 2020 also aroused widespread public concern about health; epidemics in different regions one after another in China have occupied the public view; information about the prevention and control of infectious diseases or the epidemic situation filled the website and public platforms, and diet habits and travel patterns changed, and therefore, the salt-related knowledge and behaviors status of residents may also change. WeChat is a popular social application in China with over 1 million monthly active users; about 63 million users over 55-years-old actively used WeChat on a monthly basis in 2018 from Tencent’s report. Lots of health-related information have been published on the WeChat platform, many of which are unverified or inaccurate due to the uneven quality of the publishing organizations. However, official public health WeChat accounts such as the CHLA public platform has been continuously publishing and pushing the scientific authoritative health knowledge through the platform, which can also realize the two-way interaction between the publishing organization and the recipient and can reach more people faster than offline activities. The WeChat public platform has been widely used by health education and public health institutions [31,32]. Then, these institutions could conduct related surveys through the platform to evaluate the publicity effectiveness of their activities among their target WeChat users and to further improve the quality of their work. Besides, an online survey in WeChat can save time and cost for the organizers and participants; the respondents can participate at any time of the day and it has little interference with the respondents’ daily life. To explore the status of salt-related knowledge and behaviors in 2019 and the changes 1 year later, while taking into account the active role of the WeChat platform in promoting health education and its advantages in an online investigation, we conducted a 10 day salt-related survey on an official WeChat public platform of CHLA in 2019 and 2020, separately. Salt-related knowledge, willingness and behaviors were analyzed. Scores of salt-related knowledge, salt reduction behavior and high-salt intake behavior in different groups between 2019 and 2020 were calculated and compared.

## 2. Materials and Methods

### 2.1. Data Source

The WeChat public platform of China Healthy Lifestyle for All Campaign (ID: qmjkshfsxd) was built in 2014; articles about healthy lifestyles including graphics, audio and video were published on this platform from time to time. The followers of this WeChat public platform had increased significantly year by year, and it was followed by 63,793 WeChat users on December 31 of 2018. A ten day salt-related survey was conducted on this WeChat public platform in 2019 (24 September–3 October) and 2020 (11–20 September). The online survey was approved by Chinese Center for Disease Control and Prevention Institutional Review Board (No.202108). The online survey was only published one time each year on this WeChat public platform, the link address of this survey was posted and pushed to all the followers of the platform and then forwarded to the WeChat Moments. All WeChat users can enter the survey platform through the link address to fill out the questionnaire online independently. After the survey completed, the data of participants were recorded in the form of online submission on WeChat. The survey record could not be submitted if the respondent failed to answer all questions. After submitting, the correct answers were not provided to the participant to avoid contamination of survey results between different participants. Additionally, we provided a “salt-score” (total score = 100 points) to each participant based on their record, and they could share this score with their friends from WeChat Moments, which was aimed at improving the sense of participation of respondents and to attract more WeChat users to participate in this survey.

### 2.2. WeChat-Based Survey

General information of each participant was investigated at first, including gender, age, education level, region and self-reported health status. Then, the participant was invited to complete a salt-related knowledge, willingness and behavior questionnaire. This questionnaire was designed based on the 10 core messages of salt reduction publicity week published by National Health Commission of the People’s Republic of China at 20 August 2019 [28], and the draft of the questionnaire was revised by two rounds of expert seminars. Before the questionnaire was officially posted, a small-scale population pre-survey was carried out, and the questionnaire was improved based on the feedback results to ensure that the questions were clear and understandable. Main summary of this survey can be found in Supplementary Table S1. The time taken to complete the questionnaire was automatically recorded to identify the unreasonable records. The average answering time of the participants in the pre-survey was 1.6 min, with a range of 1.1–2.8 min. 

### 2.3. Salt-Related Knowledge and Behavior Scores

Four questions were applied to assess salt-knowledge status (total scores = 13): knowledge about foods that contain salt, condiments that contain salt and should be used less, maximum daily salt intake of healthy adults and diseases that related to excessive salt intake. For question “foods that contain salt”, five foods contained salt, but no salty taste was chosen, including fine dried noodles, bread, biscuit, cheese and ice cream, and each food was scored 1 point. Soy sauce, oyster sauce, bean paste and monosodium glutamate (MSG) were chosen for the question, “Condiments that contain salt and should be used less”; each condiment scored 1 point. For the question, “Maximum daily salt intake of healthy adults”, six options were provided, including 2 g, 4 g, 5 g, 6 g, 8 g and do not know, but only the response of 5 g or 6 g can score 1 point. Three diseases, including hypertension, heart disease and stroke were presented for “Diseases that related to excessive salt intake”; each disease scored 1 point.

Six behaviors related to salt reduction (total scores = 6) were considered in this questionnaire: using salt measuring spoons, asking restaurants to use less salt, reading the sodium content on the nutrition facts table, choosing foods with low sodium content, regularly using low-sodium salt, using chili, garlic, vinegar, pepper, etc. to enhance the flavor of food and gradually reducing the amount of salt used in cooking. Participants can respond “Have this behavior” or “Did not have this behavior” for each behavior, and only people who had the behavior can score 1 point. 

High salt intake behavior (total scores = 9): frequency of using high-salt condiments such as oyster sauce, bean paste, MSG, etc., frequency of eating processed foods and canned foods, frequency of eating pickled mustard tubers, salted vegetables and sauce foods. Each behavior was classified into four levels: “Regularly”, “Occasionally”, “Rarely” and “Never”. The response of “Regularly” scored 3 points, “Occasionally” scored 2 points, “Rarely” scored 1 point and “Never” scored 0. More information about the calculation method of salt-related knowledge and behavior scores is presented in Table 1.

### 2.4. Statistical Analysis

Descriptive statistics including frequency and percentage, median and interquartile range (IQR) were conducted for general variables, salt-related knowledge and behaviors. The comparison of general information, salt-related knowledge and behaviors status in 2019 and 2020 was conducted using the chi-square test. Since salt-related knowledge and behavior scores were not of normal distribution as tested by the Kolmogorov–Smirnov test, the Wilcoxon rank sum test was used for comparing salt-related knowledge and behavior scores between 2019 and 2020. Wilcoxon rank-sum test and Kruskal–Wallis test were used for comparing the salt-related knowledge and behavior scores between subgroups. The correlation of salt-related knowledge and behavior scores was examined by Spearman correlation analysis. All statistical analyses were performed in the SPSS 22.0 software (SPSS Inc., Chicago, IL, USA), and the significance level was 0.05.

## 3. Results

### 3.1. General Information

During the survey periods, a total of 2362 records in 2019 and 14947 records were collected. After excluding the duplicate records from the same individuals or the answering time lower than 1 min or not from the mainland of China, 2109 participants’ data in 2019 and 12732 participants’ data in 2020 were left for further analysis. The general information of these participants is presented in Table 2. 

Most participants answered the questionnaire within 1 to 5 min. Among those participants in 2019, 29.8% (*n* = 629) were male, 34.7% (*n* = 732) were over 45-year-old, 8.1% (*n* = 170) had an education level of middle school and below, 40.4% (*n* = 852) came from the middle region and 11.2% (*n* = 237) were self-reported hypertension patients. Among those participants in 2020, 29.2% (*n* = 3714) were male, 39.4% ( *n*= 5021) were over 45 years old, 16.0% (*n* = 2031) had an education level of middle school and below, 19.5% (*n* = 2482) came from the middle region and 13.2% (*n* = 1677) were self-reported hypertension patients. Variables including age, education level, region, answering time and self-reported health status were significantly different between 2019 and 2020 (*p*-value < 0.05). 

In 2019, 23.4%, 52.8% and 23.8% of participants reported that they had salty, moderate and bland taste, compared to 21.8%, 53.7% and 24.4% in 2020, respectively. 88.2% of participants in 2019 had a willingness to reduce the amount of cooking salt in their households, significantly lower than 90.2% in 2020 (*p*-value < 0.05).

### 3.2. Salt-Related Knowledge

**Figure 1:**
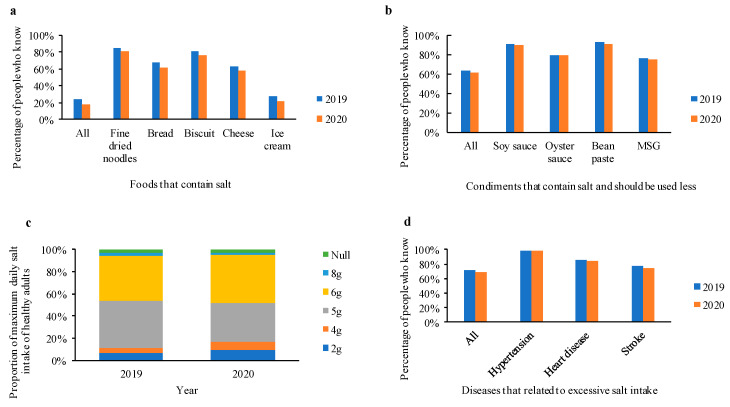
Comparison of salt-related knowledge status between 2019 and 2020. (**a**) Foods that contain salt; (**b**) condiments that contain salt and should be used less; (**c**) maximum daily salt intake of healthy adults; (**d**) diseases that related to excessive salt intake.

Results of salt-related knowledge status of participants from 2019 and 2020 are presented in Figure 1. The percentage of knowing all five foods contained salt in 2019 was 24.5%, significantly higher than 17.7% in 2020 (*p*-value < 0.05). In 2019 and 2020, over 80% of the participants knew fine dried noodles contain salt, but less than 30% knew ice cream contain salt. The percentage of those knowing fine dried noodles, bread, biscuit, cheese and ice cream contain salt in 2019 were significantly higher than those in 2020 (all *p*-value < 0.05), respectively. A number of 63.1% in 2019 and 61.5% in 2020 of participants believed all four condiments contain salt and should be used less, with no significant difference (*p*-value = 0.15). Over 78% of participants chose 5 g or 6 g for the maximum daily salt intake of healthy adults in both years. The proportion of choosing 5 g as the maximum daily salt intake of healthy adults decreased from 42.3% in 2019 to 35.4% in 2020, 40.6% and 43.1% for choosing 6 g. About 98% of participants knew that excessive salt intake would increase the risk of hypertension in 2019 and 2020. However, only 77.4% in 2019 and 74.0% in 2020 of participants knew that strokes related to excessive salt intake, with a significant difference (*p*-value < 0.05).

### 3.3. Salt-Related Behaviors

**Figure 2:**
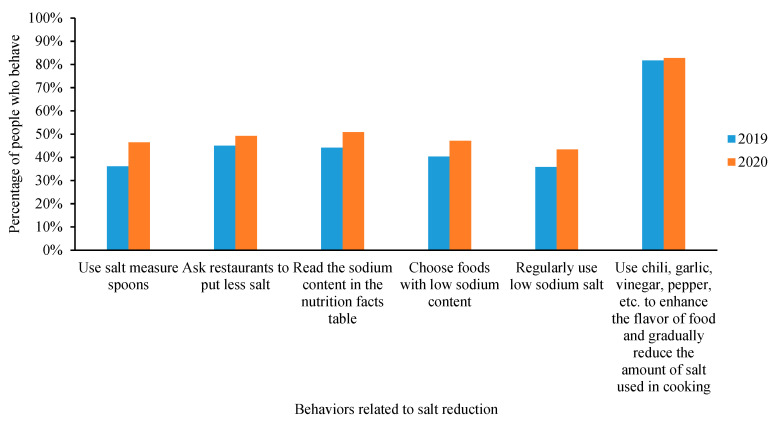
Comparison of behaviors status related to salt reduction between 2019 and 2020.

Figure 2 provides the results of salt reduction behaviors status between 2019 and 2020. The percentages of participants who used salt measuring spoons, asked restaurants to use less salt, read the sodium content on the nutrition facts table, chose foods with low sodium content and regularly used low-sodium salt, were 36.1%, 45.0%, 44.1%, 40.3% and 35.8% in 2019, and the percentage increased significantly to 46.4%, 49.2%, 50.8%, 47.1% and 43.4% in 2020 (all *p*-value < 0.05). A number of 81.7% of participants used chili, garlic, vinegar, pepper, etc. to enhance the flavor of food and gradually reduced the amount of salt used in cooking in 2019, slightly increased to 82.7% in 2020 (*p*-value = 0.057).

**Figure 3:**
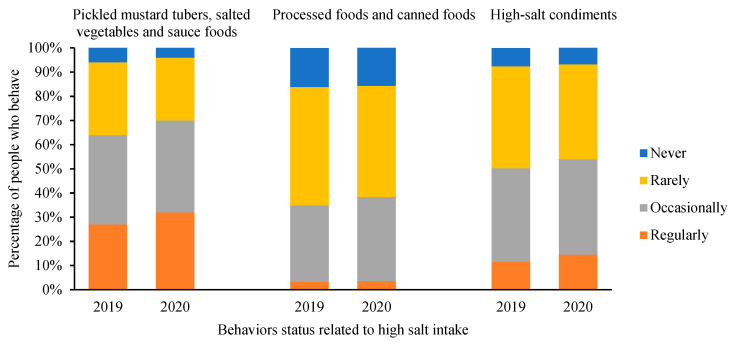
Comparison of behaviors status related to high salt intake between 2019 and 2020.

Results of behaviors status related to high salt intake between 2019 and 2020 are shown in Figure 3.

The percentage of regularly eating pickled mustard tubers, salted vegetables and sauce foods increased from 27.3% in 2019 to 31.4% in 2020, and the order of different frequencies changed from occasionally > rarely > regularly > never in 2019 to occasionally > regularly > rarely > never in 2020. Compared to 11.5% in 2019, the percentage of regularly using high-salt condiments such as oyster sauce, bean paste and MSG increased by 3% in 2020.

### 3.4. Salt-Related Knowledge and Behavior Scores

Salt-related knowledge score, salt reduction behavior score and high-salt intake behavior score were calculated separately, and the results were summarized in Table 3. The median of salt-related knowledge scores, salt reduction behavior scores and high-salt intake behavior scores were 11, 2 and 5 points in 2019 and 10, 3 and 5 points in 2020, respectively. Compared to 2019, the salt-related knowledge score was relatively lower, while the salt reduction behavior score and high-salt intake behavior score were relatively higher in 2020, and similar differences between two years still existed in age, hypertension and salt related-taste groups (all *p*-value < 0.05). 

The salt-related knowledge and behavior scores among different groups were also compared. In both 2019 and 2020, salt-related knowledge scores of females were higher than those of males, while their high-salt intake behavior scores were lower than males; salt-related knowledge and high-salt intake behavior scores in the 45-years-old-and-below group were higher, but the salt reduction behavior score was relatively lower than the over-45-years-old group; higher education level came with higher scores of salt-related knowledge and salt reduction behaviors; for participants with salty taste or without a willingness for salt reduction, salt-related knowledge and salt reduction behaviors scores were low, but the high-salt intake behavior scores were high. 

## 4. Discussion

This study found that the percentage of WeChat users who had the willingness of reducing the amount of cooking salt in their households was quite high between 2019 and 2020 in China, but the awareness level of salt-related knowledge varied greatly, similar to the result of a survey of 2444 Chinese consumers in 2017 [33]. Over 60% of participants knew all four provided condiments contain salt and should be used less or three types of diseases related to excessive salt intake, but lower than 25% of participants knew all the five foods listed contain salt in this study. There is hidden salt in foods like fine dried noodles, bread, biscuit, cheese and ice cream. Over 80% of participants knew fine dried noodles contains salt in 2019 and 2020, higher than 40.8% of knowing dragon beard noodles (a kind of fine dried noodles) contains salt in 2017 [33], but lower than 30% knew ice cream contains salt, which may be due to the sweetness and low sodium content of ice cream [34]. Unlike western countries, the main sources of sodium in China residents are cooking salt, soy sauce and other condiments; most come from home cooking [14,35]. Soy sauce, oyster sauce, bean paste and MSG are four condiments commonly used in daily life with high sodium content [34], but only less than 64% of participants believed all four condiments contain salt and should be used less. The relationship between salt intake and blood pressure has been confirmed and is widely publicized [7,28,29]; 98% of participants in this study knew that excessive salt intake would increase the risk of hypertension in 2019 and 2020, the percentages of participants who knew that excessive salt intake would increase the risk of cardiovascular diseases such as high blood pressure, heart diseases and stroke were all higher than those of Chinese consumers in 2017 [33], older rural Chinese adults in 2010 [36] and even Chinese hypertensive patients [37]. Obviously, the harmful effects of high salt intake have been widely accepted by Chinese residents.

The publicity and advocacy of the recommended maximum daily salt intake are helpful for carrying out public health interventions and salt-reduction behaviors [12,38,39]. The Dietary Guidelines of Chinese Residents 2016 and 2018 Chinese guidelines for the management of hypertension all recommended that the maximum daily salt intake of healthy adults should not exceed 6 g [13,40]. A number of 41% in 2019 and 43% in 2020 of participant knew 6 g was the maximum daily salt intake of healthy adults, close to 45.8% of Chinese hypertensive patients [37], but much higher than 6.1% in 2015 [27] and 5.0% in older rural Chinese adults in 2010 [36], credited to the advocacy and education efforts of these years. World Health Organization and the Healthy China Initiative (2019–2030) recommended that the daily salt intake of adults should not exceed 5 g [12,41], and publicity and advocacy of CHLA Campaign and Salt Reduction Publicity Week adopted this recommendation in 2019 [28]. In this study, over 78% of participants chose 5 g or 6 g for the maximum daily salt intake of healthy adults in both years, higher than 35% in Chinese consumers in 2017 [33]. Additionally, the percentage of participant identified 5 g/day in this study was higher than those in Los Angeles county residents [39] and Australia Victorian [22], but the percentage decreased from 2019 to 2020; a similar downward trend can be found in the awareness of foods that contain salt, oyster sauce should be eaten less, stroke related to excessive salt intake and the salt-related knowledge score. One possible explanation is that most publicity and education activities of salt reduction were conducted in 2019 [18,28,29], and the sudden appearance of the COVID-19 pandemic attracted public attention in 2020, leading to a decline in salt-related knowledge.

Behaviors such as using a quantitative salt spoon and reading the sodium content of food products can help people understand the amount of salt intake, then take salt constriction behaviors [19,42]. Asking restaurants to use less salt, choosing foods with low sodium content, regularly using low-sodium salt and using chili, garlic, vinegar, pepper other than salt can directly reduce the amount of salt used in cooking. Additionally, these above measures were often used or suggested in salt-reduction intervention activities [15,19,43]. The behavior rates of salt reduction of Chinese adult residents was 42.2% in 2010 and 37.3% in 2015 [27,44], and the percentage of the six chosen salt reduction behaviors were all higher than 35% in this study; over 81% of participants used chili, garlic, vinegar or pepper to enhance the flavor of food and gradually reduced the amount of salt used in cooking, and the percentage using more/other spices than salt to add flavor to food was only 42% in US adults [23], almost the half of this study, which may be due to the difference of eating habits between two countries. Additionally, the percentage of action to reduce sodium intakes such as choosing low-sodium foods or snacks, checking nutrition labels for sodium content or buying foods labeled low-sodium ranged from 34% to 36% in US adults [23], close to the result of this study. High salt intake behaviors, including regularly eating pickled mustard tubers, salted vegetables and sauce foods and using high-salt condiments, increased from 2019 to 2020, as well as the high-salt intake behaviors score, which might be related to the increase in the proportion of eating at home in 2020. For the prevention and control of the COVID-19 epidemic, more Chinese people were advised to reduce eating out and instead cook at home. Related studies reported that significant increases were observed in the frequency of consumption of preserved vegetables [45], 38.2% of Chinese residents increased their snack intake during the COVID-19 lockdown [46] and 61.6% of participants decreased eating in restaurants, while 64.8% of participants reported increased cooking at home post-lockdown [47], which may lead to an increase in the frequency of eating high-salt condiments and foods.

Females and better-educated people were more likely to have higher levels of salt-related knowledge, and they were more interested in and more active in salt reduction [21,25,33,48]. In this study, it was also found that in both 2019 and 2020, higher education levels came with higher scores of salt-related knowledge and salt reduction behaviors; females had higher salt-related knowledge scores and relatively lower high-salt intake behavior scores, but the salt reduction behavior score did not differ by gender. In most families of China, female are responsible for cooking, and they care about the healthy diet of family members, which may lead to more attention to salt-related knowledge. It was reported that better-educated people, females and young people were more knowledgeable about salt-reduction tips [33]; a similar result was found in this study, and the salt-related knowledge of the 45-years-old-and-below group was higher than those in over-45-years-old group. It should be noted that low salt-related knowledge, salt reduction score and high-salt intake behavior score were found in the hypertension patients group in this study. A previous study also found that hypertensive patients with knowledge of the recommended salt intake had a higher control rate than those without [37]. Therefore, it is necessary to carry out targeted publicity and education on salt reduction knowledge for male and hypertensive patients so as to encourage them to reduce their high-salt diet behaviors.

This study was based on the WeChat platform for all WeChat users. The subjects voluntarily participated in the online survey and could exit freely, and the results of the survey were more in line with the real situation of the participants. However, there are also several limitations in our study. First, the questionnaire was pushed to all WeChat users based on the CHLA public platform; self-reported gender, age and other information may not be as accurate as offline, face-to-face surveys, and the respondents may be more concerned about lifestyle and dietary nutrition than the general population, and extrapolation of the conclusions of this study to the entire population might be limited. However, these participants who actively interested in this survey are targeted residents of the CHLA Campaign publicity and intervention. The results revealed the weak publicity knowledge points and intervention groups of the CHLA Campaign, and could provide a reference for setting the salt reduction action goal in the future. Second, we analyzed the change of salt-related knowledge and behavior between 2019 and 2020 based on two cross-sectional surveys, but did not follow up with the same participants; changes of participants during the 2-year period cannot be observed. Additionally, the total number of responders in 2020 was much higher than those in 2019, which might be caused by the COVID-19 pandemic, the theme activity of Healthy Lifestyle Promotion Month in early September and the increasing followers of our WeChat public platform, and the sample bias of the comparison results in this study may exist, which would make our study less persuasive. Third, attitude-related questions such as reasons for a reluctance to salt reduction, responsibility for salt reduction and preference of salt-reduction measures, which were often used in previous articles [21,22,33] may be helpful for formulating salt reduction policies and strategies; in this study, we only asked salt-related taste and willingness of reducing the amount of cooking salt in households, which may fail to fully reflect participants’ attitudes towards salt reduction and analyze the inner relationship of salt-related Knowledge–Attitude–Practice (KAP). Besides, the self-reported salt related behaviors of participants were not verified by urine assessment of sodium intake; the actual change in the salt intake of these WeChat users was unknown.

## 5. Conclusions

In order to dynamically understand the public’s awareness and practice of the salt reduction core information released by the CHLA public platform and to select targeted and effective salt reduction interventions for the CHLA Campaign, a WeChat-based online questionnaire was established. The status and changes of salt-related knowledge and behaviors of Chinese WeChat users between 2019 and 2020 were analyzed. Results show that the willingness of reducing the amount of cooking salt in households was very high, but the level of salt-related knowledge decreased and the proportion of salt reduction and high-salt intake behaviors increased from 2019 to 2020, which may due to the potential impact of COVID-19 epidemic. The distribution of salt-related knowledge and behaviors between subgroups was basically indifferent within 2 years. The results also point out that promotion and education of salt-related knowledge and online behavior intervention are still needed, particularly for male and hypertension patients in the future, which will be fully considered in CHLA Campaign salt reduction action. According to the results, the national CHLA office has produced new video materials and standard education series courseware relate to salt reduction and graphic materials on low-salt diet strategies for people with hypertension or cardiovascular diseases. Additionally, we have integrated these existing salt reduction materials and developed a WeChat mini-program for WeChat users’ online intervention, which will be used in the next step to promote the national salt reduction action. In addition, we are also adjusting the content and long-term goals of the upcoming salt-reduction action plan based on the survey results. We believe that this WeChat-based survey will provide basic data for the follow-up investigation, which we will definitely continue to conduct, and it might be a useful instrument for other salt-reduction activities.

## Figures and Tables

**Table 1:** The calculation method of salt-related knowledge and behavior scores.

	Questions	Score	Setting
Salt-related knowledge score (total scores = 13)	Foods that contain salt	5	Fine dried noodles = 1, Bread = 1, Biscuit = 1, Cheese = 1, Ice cream = 1
	Condiments that contain salt and should be used less	4	Soy sauce = 1, Oyster sauce = 1, Bean paste = 1, MSG = 1
	Maximum daily salt intake of healthy adults	1	5 g or 6 g = 1
	Diseases that related to excessive salt intake	3	Hypertension = 1, Heart disease = 1, Stroke = 1
Salt-reduction behavior score (total scores = 6)	Use salt measuring spoons	1	Have this behavior = 1
	Ask restaurants to use less salt	1	Have this behavior = 1
	Read the sodium content on the nutrition facts table	1	Have this behavior = 1
	Choose foods with low sodium content	1	Have this behavior = 1
	Regularly use low-sodium salt	1	Have this behavior = 1
	Use chili, garlic, vinegar, pepper, etc. to enhance the flavor of food and gradually reduce the amount of salt used in cooking	1	Have this behavior = 1
High salt intake behavior score (total scores = 9)	Frequency of using high-salt condiments such as oyster sauce, bean paste, MSG, etc.	3	Regularly = 3, Occasionally = 2, Rarely = 1, Never = 0
	Frequency of eating processed foods and canned foods	3	Regularly = 3, Occasionally = 2, Rarely = 1, Never = 0
	Frequency of eating pickled mustard tubers, salted vegetables and sauce foods	3	Regularly = 3, Occasionally = 2, Rarely = 1, Never = 0

**Table 2:** General information.

Variables	Year 2019 (*N* = 2109)		Year 2020 (*N* = 12,732)		χ^2^	*p*-Value
	*N*	%	*N*	%		
**Gender**					0.374	0.541
Male	629	29.8	3714	29.2		
Female	1480	70.2	9018	70.8		
**Age**					17.036	<0.001
≤45	1377	65.3	7711	60.6		
>45	732	34.7	5021	39.4		
**Education level**					251.467	<0.001
Middle school and below	170	8.1	2031	16.0		
High school	556	26.4	4214	33.1		
College	1177	55.8	5968	46.9		
Postgraduate	206	9.8	519	4.1		
**Region**					477.862	<0.001
East	764	36.2	5393	42.4		
Middle	852	40.4	2482	19.5		
West	493	23.4	4857	38.1		
**Answering time**					61.155	<0.001
≥1–2min	769	36.5	3714	29.2		
≥2–5 min	1263	59.9	8195	64.4		
≥5 min	77	3.7	823	6.5		
**Self-reported health status**						
Hypertension	237	11.2	1677	13.2	6.024	0.014
Basic health	1826	86.6	10790	84.7	4.776	0.029
**Salt-related taste**					2.645	0.266
Salty	494	23.4	2781	21.8		
Moderate	1113	52.8	6842	53.7		
Bland	502	23.8	3109	24.4		
**Willingness for salt reduction**					9.163	0.027
Yes	1861	88.2	11487	90.2		
No	130	6.2	694	5.5		
Do not care	102	4.8	485	3.8		
Do not know	16	0.8	66	0.5		

**Table 3:** Distribution of salt-related knowledge and behavior scores in 2019 and 2020 (Median (IQR)).

Variables	Salt-Related Knowledge Score				Salt Reduction Behavior Score				High-Salt Intake Behavior Score			
	2019	2020	Z	*p*-Year	2019	2020	Z	*p*-Year	2019	2020	Z	*p*-Year
**Total**	11(3)	10(4)	6.908	<0.001	2(3)	3(4)	−8.132	<0.001	5(3)	5(2)	−4.947	<0.001
Gender												
Male	10(4)	10(3)	4.391	<0.001	2(3)	3(4)	−5.784	<0.001	5(3)	5(2)	−1.814	0.070
Female	11(3)	10(4)	5.431	<0.001	3(3)	3(4)	−5.917	<0.001	5(3)	5(2)	−4.761	<0.001
*p*-group	0.001	<0.001			0.173	0.137			0.013	0.001		
Age												
≤45	11(3)	10(4)	6.157	<0.001	2(3)	3(4)	−5.857	<0.001	5(3)	5(2)	−4.029	<0.001
>45	10(4)	10(4)	2.730	0.006	3(3)	4(3)	−5.035	<0.001	4(3)	5(3)	−3.458	<0.001
P-group	<0.001	<0.001			<0.001	<0.001			<0.001	<0.001		
Education level												
Middle school and below	8(4)	9(5)	−2.003	0.045	2(3)	3(3)	−5.746	<0.001	4(3)	5(3)	−2.779	0.005
High school	9(4)	10(3)	−1.447	0.148	3(4)	3(3)	−5.445	<0.001	5(3)	5(3)	−2.467	0.014
College	11(3)	11(3)	4.123	<0.001	2(4)	3(4)	−3.621	<0.001	5(3)	5(2)	−4.112	<0.001
Postgraduate	12(2)	11(3)	2.906	0.004	3(3)	3(3)	0.285	0.776	5(3)	5(2)	−1.247	0.212
*p*-group	<0.001	<0.001			0.028	<0.001			0.072	<0.001		
Region												
East	11(3)	10(4)	6.890	<0.001	3(4)	4(3)	−6.342	<0.001	5(3)	5(2)	−2.891	0.004
Middle	10(4)	10(4)	−1.379	0.168	2(3)	3(4)	−3.195	0.001	5(3)	5(3)	−1.820	0.069
West	11(4)	10(3)	5.263	<0.001	2(3)	3(4)	−4.055	<0.001	5(3)	5(2)	−2.729	0.006
*p*-group	<0.001	<0.001			<0.001	<0.001			0.535	<0.001		
Answering time												
≥1–2min	11(4)	11(3)	5.383	<0.001	2(3)	3(4)	−2.192	0.0284	5(3)	5(2)	−3.939	<0.001
≥2–5 min	10(4)	10(4)	2.681	0.007	3(4)	3(3)	−7.134	<0.001	5(3)	5(3)	−3.853	<0.001
≥5 min	9(4)	9(4)	2.012	0.044	2(4)	4(3)	−2.864	0.004	5(3)	5(3)	−0.563	0.574
*p*-group	<0.001	<0.001			0.736	<0.001			0.051	<0.001		
Hypertension												
Yes	10(4)	10(4)	2.007	0.045	2(3)	3(3)	−4.445	<0.001	5(2)	5(3)	−2.049	0.041
No	11(3)	10(4)	6.519	<0.001	3(3)	3(4)	−6.989	<0.001	5(3)	5(2)	−4.398	<0.001
*p*-group	0.019	<0.001			0.468	0.002			0.011	<0.001		
Salt-related taste												
Salty	11(4)	10(4)	3.143	0.002	2(2)	2(3)	−3.452	<0.001	6(2)	6(2)	−2.216	0.027
Moderate	11(3)	10(4)	4.120	<0.001	2(3)	3(3)	−6.826	<0.001	5(3)	5(2)	−4.160	<0.001
Bland	11(3)	10(4)	5.048	<0.001	4(3)	4(4)	−3.520	<0.001	4(2)	4(2)	−3.530	<0.001
*p*-group	0.011	<0.001			<0.001	<0.001			<0.001	<0.001		
Willingness for salt reduction												
Yes	11(3)	10(4)	7.009	<0.001	3(4)	3(3)	−7.276	<0.001	5(3)	5(2)	−5.208	<0.001
No	9(4)	9(5)	1.929	0.054	1(1)	1(1)	−1.422	0.155	5(3)	6(3)	−0.897	0.370
*p*-group	<0.001	<0.001			<0.001	<0.001			<0.001	<0.001		

Note: *p*-year is the *p*-value of Wilcoxon rank-sum test for comparing salt-related knowledge and behavior scores between 2019 and 2020. Note: *p*-group is the *p*-value of the comparison of salt-related knowledge and behavior scores between different groups. Wilcoxon rank-sum test was used for gender, age and hypertension. Kruskal–Wallis test was used for education level, region, answering time, salt-related taste and willingness for salt reduction.

## Data Availability

The data presented in this study are available on request from the corresponding author.

## Supplementary Materials

The following are available online at https://www.mdpi.com/article/10.3390/nu13072141/s1, Table S1: Main summary of the salt WeChat-based survey.
