# Supplementary material for: Comparison of Salt-Related Knowledge and Behaviors Status of WeChat Users between 2019 and 2020
Source: Nutrients. 2021 Jun 22;13(7):2141. doi: 10.3390/nu13072141 (PMC8308297; doi:10.3390/nu13072141)
Supplement: Supplementary file 1 [file nutrients-13-02141-s001.zip › nutrients-1232812-supplementary.pdf]

Supplementary Table S1. Main summary of the salt WeChat-based survey

	Questions	Response(s)
General Information.	What is your gender?	Male, Female
General Information.	How old are you?	≤45, >45
General Information.	What is your education level?	Middle school and below, High school, College, Postgraduate
General Information.	Where are you from?	Specific province, else
General Information.	What is your health status?	Hypertension, Basic health
General Information.	What is your salt-related taste?	Salty, Moderate, Bland
General Information.	Do you have the willingness of reducing the amount of cooking salt in your households	Yes, No, Don’t care, Don’t know
Salt-related knowledge	Do you know which of the following foods contain salt? (multiple choice)	Fine dried noodles=1, Bread=1, Biscuit=1, Cheese=1, Ice cream=1, , Don’t know
Salt-related knowledge	From the perspective of salt control, which of the following condiments do you believe that contain salt and should be used less? (multiple choice)	Soy sauce=1, Oyster sauce=1, Bean paste =1, MSG=1, Don’t know
Salt-related knowledge	Do you know how many grams of salt for healthy adults should not exceed daily?	2g, 4g, 5g, 6g, 8g, Don’t know
Salt-related knowledge	Diseases that related to excessive salt intake(multiple choice)	Hypertension, Heart disease, Stroke, , Don’t know
Salt reduction behavior	Do you use salt measure spoons?	Yes, No
Salt reduction behavior	When dining out, do you ask restaurants to put less salt?	Yes, No
Salt reduction behavior	When buying packaged food, do you read the sodium content in the nutrition facts table?	Yes, No
Salt reduction behavior	Do you choose foods with low "sodium" content in the nutrition ingredient list?	Yes, No
Salt reduction behavior	Do you regularly use low sodium salt?	Yes, No
Salt reduction behavior	Do you use chili, garlic, vinegar, pepper, etc. to enhance the flavor of food and gradually reduce the amount of salt used in cooking?	Yes, No
High salt intake behavior	Do you use high-salt condiments such as oyster sauce, bean paste, MSG, etc. in daily life?	Regularly use, Occasionally use, Rarely use, Never use
High salt intake behavior	Do you eat processed foods and canned foods in daily life?	Regularly eat, Occasionally eat, Rarely eat, Never eat
High salt intake behavior	Do you eating pickled mustard tubers, salted vegetables and sauce foods in daily life?	Regularly eat, Occasionally eat, Rarely eat, Never eat
